# Effect of TNF-α on osteocyte RANKL expression during orthodontic tooth movement

**DOI:** 10.1016/j.jds.2021.03.006

**Published:** 2021-03-31

**Authors:** Aseel Marahleh, Hideki Kitaura, Fumitoshi Ohori, Takahiro Noguchi, Yasuhiko Nara, Adya Pramusita, Ria Kinjo, Jinghan Ma, Kayoko Kanou, Itaru Mizoguchi

**Affiliations:** Division of Orthodontics and Dentofacial Orthopedics, Department of Translational Medicine, Tohoku University Graduate School of Dentistry, Sendai, Japan

**Keywords:** Orthodontic tooth movement, Osteoclast, Osteocyte, RANKL, TNF-α

## Abstract

**Background/purpose:**

Orthodontic tooth movement (OTM) is facilitated by two events; bone resorption on the compression side and bone formation on the tension side simultaneously termed bone remodeling. Osteocytes play a critical role in bone remodeling during OTM, as they have been described as the critical source of nuclear factor-κB ligand (RANKL) necessary for bone remodeling during OTM. Tumor necrosis factor (TNF)-α is a cytokine that acts by amplifying RANKL expression in osteocytes. In this study, we evaluated the effects of TNF-α on RANKL expression in osteocyte during OTM.

**Materials and methods:**

We assessed whether TNF-α influenced RANKL expression in osteocyte during orthodontic tooth movement by using wild-type (WT) and TNF receptor I and II deficient (TNFRsKO) mice. A Nickel-titanium closed coil spring was attached to the maxillary alveolar bone near the incisors and the upper left first molar, and the first molars were moved mesially in WT and TNFRsKO mice. After OTM, the number of RANKL-positive osteocytes in the alveolar bone was evaluated by immunohistochemistry.

**Results:**

The number of RANKL-positive osteocyte in the alveolar bone significantly increased in WT mice than in TNFRsKO mice after OTM.

**Conclusion:**

The results indicate that TNF-α induces the expression of RANKL in osteocyte during OTM.

## Introduction

Osteoclasts derived from hematopoietic stem cells are the chief bone resorbing cell which mediate bone remodeling.^1^ Macrophage-colony-stimulating factor (M-CSF) and receptor activator of nuclear factor κB ligand (RANKL) have been found to be essential factors of osteoclast formation.^2^ Tumor necrosis factor (TNF)-α, a principal pro-inflammatory cytokine was also found to be inductive of osteoclast differentiation.^3^^,^^4^ TNF-α regulates osteoclast formation in erosive bone diseases such as rheumatoid arthritis and periodontal diseases where there is an immoderate presence of osteoclasts leading to excessive bone resorption.^5^

Osteocytes are terminally differentiated osteoblasts which are entombed in their secreted bone matrix.^6^^,^^7^ Osteocytes reside in bone spaces termed lacunae and account for 90% of the bone cell population. Osteocytes communicate through an extravagant canalicular system connecting osteocyte lacunae to each other, to other cell types, and to the bone surface.^8^ This complex communication apparatus allows the osteocyte to function as a mechanosensory cell, to remodel its perilacunar matrix and to regulate mineral metabolism.^9^ RANKL expressed by osteocytes has been reported to play an important role in bone metabolism, as RANKL-deficient mice developed an osteopetrotic phenotype that became increasingly apparent with age. It has been argued recently that osteocyte-secreted RANKL is the most valuable for physiologically driven osteoclast formation in the remodeling bone.^10^^,^^11^ Furthermore, RANKL-deficient mice have been shown to be protected against bone loss resulting from mechanical force unloading.^11^ Moreover, negating osteocyte RANKL prevents infection-induced periodontal bone loss.^12^ Conditional loss of RANKL expression in osteocytes led to an increase of cancellous bone mass in an osteogenesis imperfecta mouse model.^13^ It is apparent that osteocyte RANKL strongly affects bone resorption both in physiological and pathological bone events.

TNF-α-induced osteoclast formation is a major contributor to bone destruction in disorders such as rheumatoid arthritis and periodontal disease.^14^^,^^15^ Many cytokines employ their function on the osteocyte by amplifying their osteoclast formation and bone resorption ability, TNF-α is one such cytokine. The osteocyte also secretes a variety of cytokines and signaling molecules which affect neighboring osteocytes, other bone cells such as osteoblasts and osteoclasts, immune system cells around the bone matrix and other distant organs, which mean osteocytes exert their functions through paracrine, autocrine, and endocrine routes.^16^ We previously reported that TNF-α directly increases RANKL expression in osteocytes as well as induces osteoclast formation both *in vitro* and *in vivo*.^17^

Orthodontic tooth movement counts on remodeling of the alveolar bone through an external mechanical force. The external force induces growth factor, cytokine, and neurotransmitter release, which signals to enhance osteoclastogenesis with compressive forces and osteoblast formation with tension, leading to the coupling of bone resorption and bone formation which are prerequisites of bone remodeling.^18^^,^^19^ Forces applied to the compression side of the periodontal ligament induce osteoclast formation and resorption of the alveolar bone, resulting in tooth movement.^18^ It has been reported that TNF-α is expressed at the compression side in the periodontal ligament and play a crucial role in the regulation of tooth movement and osteoclast formation during OTM.^20^^,^^21^

The role of osteocytes in OTM is revealed in transgenic mice which osteocytes express the diphtheria toxin receptor under the control of the Dmp1-promoter. After diphtheria toxin injection to these mice, OTM was hindered when compared to wild type mice. Both tooth movement and osteoclast formation decreased on the compression side.^22^ Another recent report showed that using mice specifically lacking RANKL in osteocytes decreased bone remodeling and OTM.^23^ In our recent study, TNF-α enhanced sclerostin expression in osteocytes and subsequently sclerostin enhanced RANKL expression in osteocytes.^24^ This has been shown to be beneficial to OTM and bone remodeling. However, the direct effect of TNF-α on osteocytes RANKL expression during orthodontic tooth movement is still to be clarified.

In this study, we evaluated the effect of TNF-α on the expression of RANKL in osteocytes during OTM by using TNF receptor I and II deficient (TNFRs KO) mice.

## Materials and methods

### Experimental animals

Male C57BL6/J mice were purchased from (CLEA, Tokyo, Japan) and TNFRs KO mice (Tnfrsf1a^*tm1lmx*^Tnfrsf1b^*tm1lmx*^) were obtained from (The Jackson Laboratory, Bar Harbor, ME, USA) aged eight to ten-week-old. The mice were maintained with a 12/12-h light/dark cycle at 21–24 °C. The mice were provided with a granular diet (Oriental Yeast, Tokyo, Japan) to ease eating during OTM without the use of excessive chewing force.

All experimental procedures conformed to the Regulations for Animal Experiments and Related Activities at Tohoku University, and were reviewed and approved by the Institutional Laboratory Animal Care and Use Committee of Tohoku University, and finally approved by the President of Tohoku University.

### Experimental orthodontic tooth movement in mice

**Figure 1:**
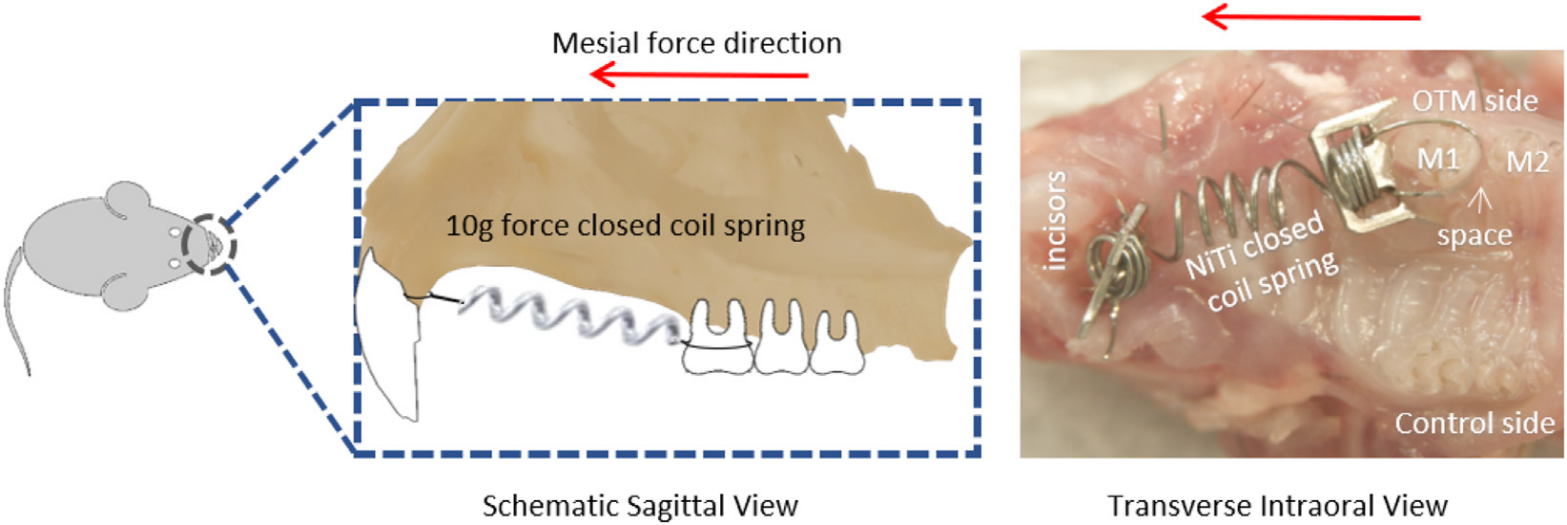
Schematic diagram of orthodontic tooth movement in mice and intraoral image of a nickel-titanium closed-coil spring attached between the maxillary alveolar bone underneath the incisors and the upper-left first molar. The appliance was tied with a 0.1 mm stainless steel wire through holes made by drilling into the alveolar bone, and also tied to the first molar at the posterior end. The resultant force generated mesial movement of the maxillary left first molar.

For the application of orthodontic tooth movement, mice were put under anesthesia, and a nickel-titanium (Ni–Ti) closed coil spring (Tomy, Fukushima, Japan) was hooked to the left maxillary first molar, and the maxillary alveolar bone underneath the incisors, which resulted in a 10 g force according to manufacturer's instructions moving the maxillary left first molar in a mesial direction. For appliance fixation, a hole was made by drilling with a slow-speed handpiece and a tungsten carbide bur underneath each of the two incisors at the alveolar bone level; then the appliance was tied to a 0.1 mm stainless steel wire through this hole, this facilitated the secure attachment of the appliance to both the left maxillary first molar and the alveolar bone (Fig. 1).^25^, ^26^, ^27^ Each group contained four mice and orthodontic tooth movement was carried out for 12 days.

### Preparation of samples for histological observation

The mice were sacrificed by an overdose of 5% isoflurane in an inhalation chamber. The maxillae were cut off from the skull and fixed in 4% paraformaldehyde at 4 °C overnight. Maxillae were decalcified in 14% EDTA for 3 days at 4 °C. Decalcified maxillae were dehydrated in graded ethanol and embedded in paraffin blocks. The samples were cut into horizontal sections at a thickness of 4 μm from a starting point of approximately 150 μm from the root furcation of the maxillary left first molar as described previously.^25^, ^26^, ^27^ The deparaffinized sections were stained for tartrate-resistant acid phosphatase (TRAP) activity and counterstained with hematoxylin. TRAP staining was carried out using Fast Red Violet LB Salt (MO, USA, Sigma–Aldrich), Naphthol-ASMX-phosphate (MO, USA, Sigma–Aldrich), and 50 mM sodium tartrate. For RANKL staining, the sections were washed with 3% H_2_O_2_ in PBS for 15 min and treated with 3% skim milk for 30 min at 37 °C for blocking. The treated sections were treated with anti-RANKL antibody (FL-317 SCBT rabbit polyclonal IgG) 1:50 in Can Get Immunostain solution B (Toyobo, Osaka, Japan) overnight at 4 °C for immunohistochemistry. After washing, the sections were treated with Histofine® Simple Stain™ Mouse MAX PO (R) (Nichirei Bioscience, Tokyo, Japan) at room temperature for 1 h. The sections were counterstained with hematoxylin. The number of RANKL-positive osteocytes at the alveolar bone on the compression side of the distobuccal root on day 6 and on day 12 during OTM was evaluated.

### Statistical analysis

All data are presented as the mean ± standard deviation of independent biological replicas. Statistical analysis was performed using Scheffe's F-test. Statistical significance was assumed at a threshold of p < 0.05.

## Results

### TNF-α enhances RANLK expression in osteocyte during OTM

**Figure 2:**
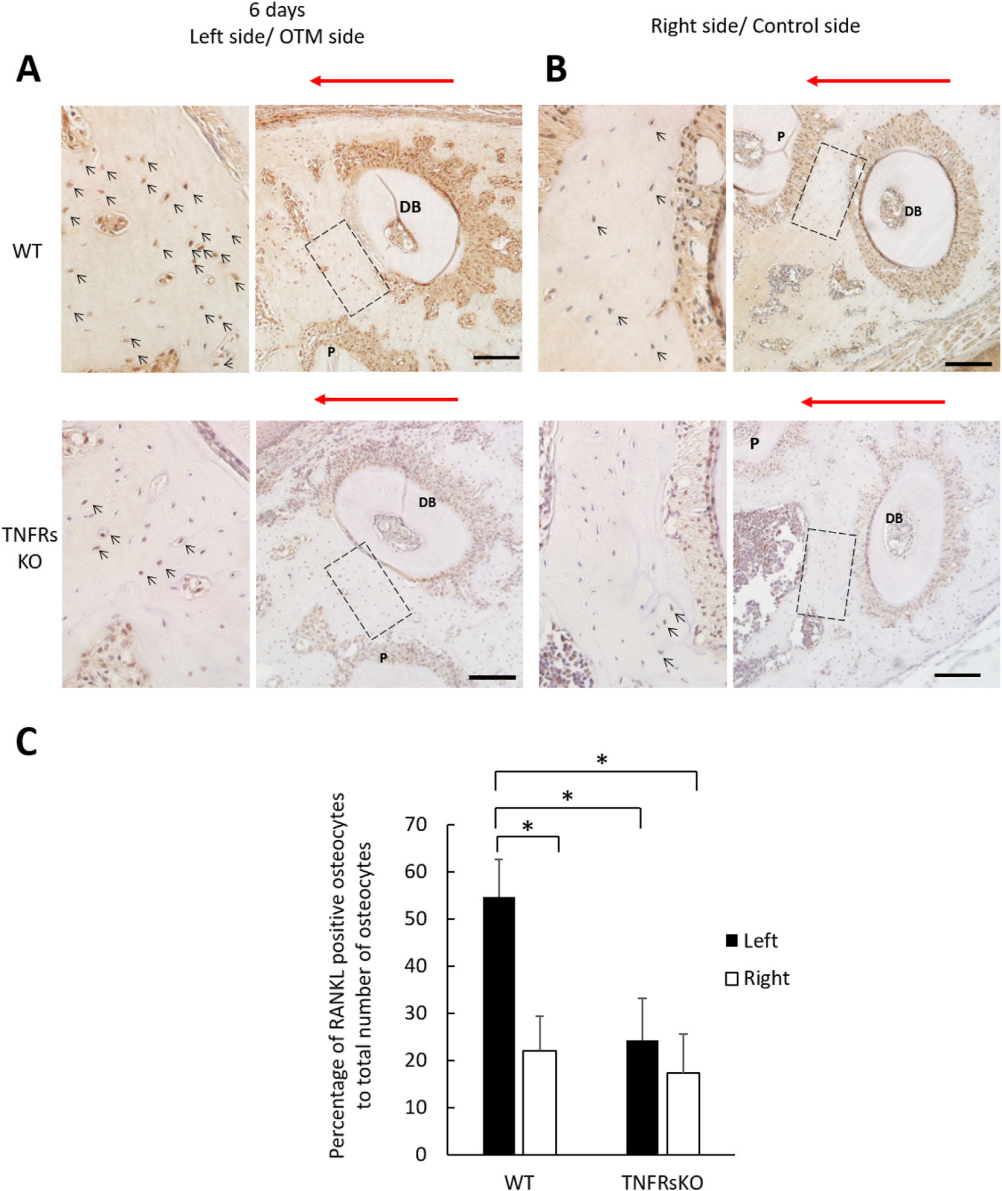
Effect of TNF-α on RANKL expression in osteocytes during OTM of 6 days. (A) and (B) Histological images of the disto-buccal root of the maxillary left and right first molars after OTM in each group. The samples were treated with anti-RANKL antibodies and counterstained with hematoxylin. (C) Percentage of RANKL-positive osteocytes to total number of osteocytes located in the alveolar bone near the compression/mesial side of the distobuccal root after OTM as indicated per group. Red arrows: direction of orthodontic force; arrow heads: RANKL-positive osteocytes; DB: disto-buccal root; P: palatal root. Scale bars = 100 μm. Data are shown as the means ± standard deviation. Statistical significance was evaluated by Scheffe's test (n = 4, ∗P < 0.01).

**Figure 3:**
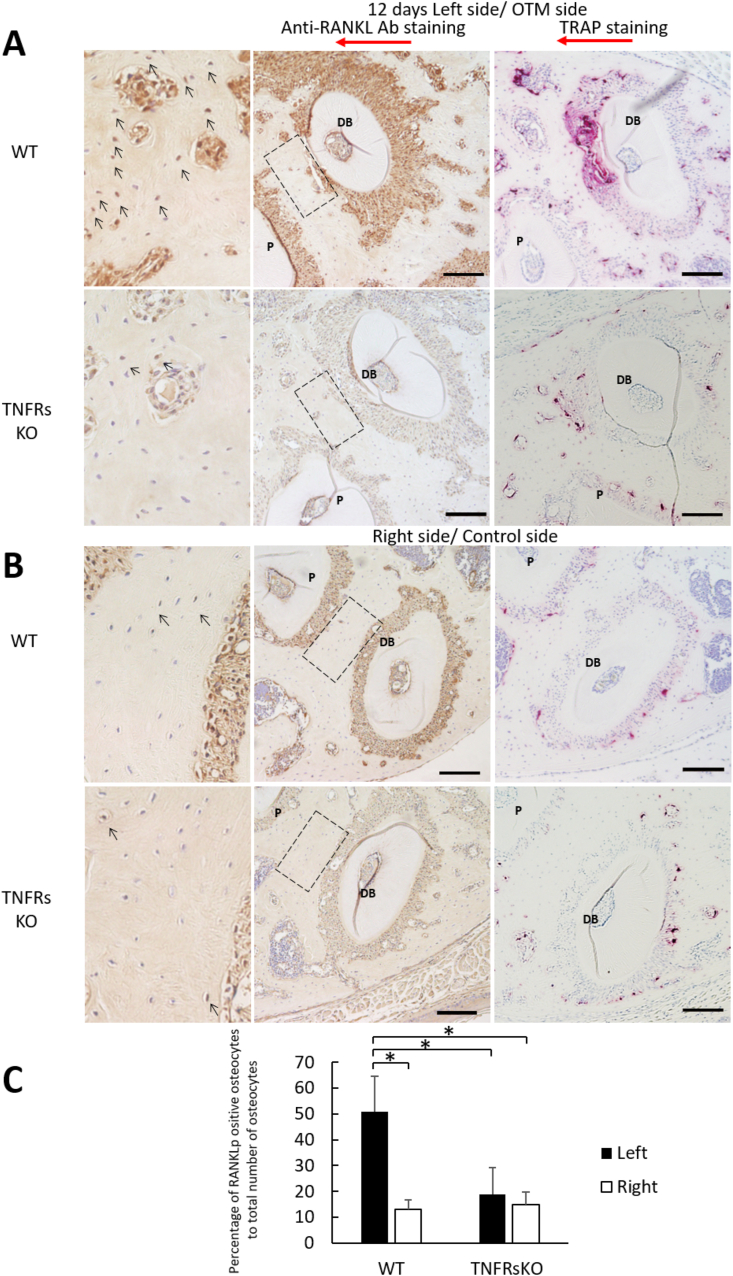
Effect of TNF-α on RANKL expression in osteocytes during OTM of 12 days. (A) and (B) Histological image of the disto-buccal root of the maxillary left and right first molars after OTM in each group. The samples were treated with anti-RANKL antibodies and counterstained with hematoxylin. The right side image of each A and B panels represent TRAP stained images of the bone around the disto-buccal root of the maxillary left and right first molars. (C) Percentage of RANKL-positive osteocytes located in the alveolar bone near the compression/mesial side of the distobuccal root after OTM as indicated per group. Red arrows: direction of orthodontic force; arrow heads: RANKL-positive osteocytes; DB: disto-buccal root; P: palatal root. Scale bars = 100 μm. Data are shown as the means ± standard deviation. Statistical significance was evaluated by Scheffe's test (n = 4, ∗P < 0.01).

After 6 days and 12 days of orthodontic tooth movement in mice, the mice were sacrificed, and the maxillae were prepared for immunohistochemical analysis. After 6 and 12 days of OTM there was significantly a fewer number of RANKL-positive osteocytes in the alveolar bone at the compression side of the distobuccal root in TNFRsKO mice when compared to WT mice, however there was no appreciable difference at the opposite right control (non-OTM) side (Figure 2, Figure 3). Thus, OTM-mediated RANKL expression in osteocytes experienced a decrease in TNFRsKO mice.

## Discussion

In the present study, we showed that RANKL expression increased in WT mice, but not TNFRsKO mice during OTM. We found that TNF-α stimulated RANKL expression in osteocytes during OTM. The results suggested that orthodontic force mediated-TNF-α release is a significant factor for facilitating the expression of RANKL in osteocytes. This is the first experiment to examine an interaction between TNF-α stimulation and RANKL expression in osteocytes during OTM.

We previously reported that TNF-α injection to supracalvariae of mice induced osteoclast formation in calvaria *in vivo.*^28^^,^^29^ Furthermore, we showed that TNF-α enhanced RANKL expression in the cells of supracalvariae through measuring mRNA level of RANKL after TNF-α injection to the supracalvariae of mice.^28^^,^^29^ Recently, we tested TNF-α-induced RANKL expression in osteocytes using immunohistochemistry. The results showed that RANKL osteocyte expression increased in TNF-α-injected mice, which means that TNF-α increases RANKL expression in osteocytes *in vivo.*

A recent report where mice lacking RANKL in osteocyte were used reported that these mice experienced a severe osteopetrotic phenotype as they aged. The results considered that osteocyte RANKL is most relevant to physiological bone remodeling.^10^ Furthermore, osteocytes also were shown to express RANKL in pathological condition, leading to bone destruction in diseases such as inflammatory bowel diseases and periodontal diseases.^30^^,^^31^ In one of our studies, we examined the direct effect of TNF-α on osteocytes. The results showed that TNF-α directly enhances RANKL expression as well as osteocyte-induced osteoclast formation both *in vitro* and *in vivo.*^17^ It has been shown that TNF-α increased in human gingival crevicular fluid during OTM.^32^^,^^33^ Our previous reports studied the link between TNF-α mediated osteoclast formation and OTM using TNFRsKO mice.^20^^,^^21^ And in this study, we assessed the effect of TNF-α on inducing RANKL expression in osteocytes during OTM by using TNFRsKO mice. We evaluated RANKL expression by applying orthodontic force to the upper-left first molar which resulted in a mesial force direction for 6 days and 12 days using immunohistochemistry. RANKL-positive osteocytes in TNFRsKO mice were significantly lower than that in WT mice both on day 6 and day 12. Our experiments also confirmed previously published results showing that TNF-α is also important for osteoclastogenesis during OTM as TNFRsKO mice experienced less osteoclast formation than that in WT mice.^20^^,^^21^

In our pervious study, we analyzed target cell contribution to the TNF-α pool and subsequent osteoclast formation during OTM using TNFRsKO and WT chimeric mice. The chimeric mice were generated by transplanting the marrow of either TNFRsKO or WT mice into lethally irradiated WT and TNFRsKO mice, resulting in four mice types. The results showed that TNF-α-responsive stromal cells (including osteocytes) in mice that were transplanted with TNFRsKO marrow are most contributing to osteoclast formation in OTM setting.^25^ These findings suggested that TNF-α-responsive osteocytes may contribute to osteoclast formation in OTM. In the present study, TNF-α expressed as a result of mechanical stress was responsible for osteocytes expression of RANKL. These findings suggested that TNF-α-responsive osteocytes are important cells for osteoclast formation during OTM.

## Declaration of Competing Interest

The authors have no conflicts of interest relevant to this article.

## References

[1] Ponzetti M., Rucci N. (2019). Updates on osteoimmunology: what's new on the cross-talk between bone and immune system. Front Endocrinol.

[2] Teitelbaum S.L. (2007). Osteoclasts: what do they do and how do they do it?. Am J Pathol.

[3] Azuma Y., Kaji K., Katogi R., Takeshita S., Kudo A. (2000). Tumor necrosis factor-alpha induces differentiation of and bone resorption by osteoclasts. J Biol Chem.

[4] Kobayashi K., Takahashi N., Jimi E. (2000). Tumor necrosis factor alpha stimulates osteoclast differentiation by a mechanism independent of the ODF/RANKL-RANK interaction. J Exp Med.

[5] Zhao B. (2017). TNF and bone remodeling. Curr Osteoporos Rep.

[6] Franz-Odendaal T.A., Hall B.K., Witten P.E. (2006). Buried alive: how osteoblasts become osteocytes. Dev Dynam.

[7] Holmbeck K., Bianco P., Pidoux I. (2005). The metalloproteinase MT1-MMP is required for normal development and maintenance of osteocyte processes in bone. J Cell Sci.

[8] Bonewald L.F. (2007). Osteocytes as dynamic multifunctional cells. Ann N Y Acad Sci.

[9] Bonewald L.F. (2011). The amazing osteocyte. J Bone Miner Res.

[10] Nakashima T., Hayashi M., Fukunaga T. (2011). Evidence for osteocyte regulation of bone homeostasis through RANKL expression. Nat Med.

[11] Xiong J., Piemontese M., Onal M. (2015). Osteocytes, not osteoblasts or lining cells, are the main source of the RANKL required for osteoclast formation in remodeling bone. PloS One.

[12] Graves D.T., Alshabab A., Albiero M.L. (2018). Osteocytes play an important role in experimental periodontitis in healthy and diabetic mice through expression of RANKL. J Clin Periodontol.

[13] Zimmerman S.M., Heard-Lipsmeyer M.E., Dimori M. (2018). Loss of RANKL in osteocytes dramatically increases cancellous bone mass in the osteogenesis imperfecta mouse (oim). BoneKEy Rep.

[14] Redlich K., Hayer S., Ricci R. (2002). Osteoclasts are essential for TNF-alpha-mediated joint destruction. J Clin Invest.

[15] Graves D.T., Cochran D. (2003). The contribution of interleukin-1 and tumor necrosis factor to periodontal tissue destruction. J Periodontol.

[16] Kitaura H., Marahleh A., Ohori F. (2020). Osteocyte-related cytokines regulate osteoclast formation and bone resorption. Int J Mol Sci.

[17] Marahleh A., Kitaura H., Ohori F. (2019). TNF-α directly enhances osteocyte RANKL expression and promotes osteoclast formation. Front Immunol.

[18] Kitaura H., Kimura K., Ishida M. (2014). Effect of cytokines on osteoclast formation and bone resorption during mechanical force loading of the periodontal membrane. Sci World J.

[19] Sato T., Miyazawa K., Suzuki Y. (2014). Selective β2-adrenergic antagonist butoxamine reduces orthodontic tooth movement. J Dent Res.

[20] Kitaura H., Yoshimatsu M., Fujimura Y. (2008). An anti-c-Fms antibody inhibits orthodontic tooth movement. J Dent Res.

[21] Yoshimatsu M., Shibata Y., Kitaura H. (2006). Experimental model of tooth movement by orthodontic force in mice and its application to tumor necrosis factor receptor-deficient mice. J Bone Miner Metabol.

[22] Matsumoto T., Iimura T., Ogura K., Moriyama K., Yamaguchi A. (2013). The role of osteocytes in bone resorption during orthodontic tooth movement. J Dent Res.

[23] Shoji-Matsunaga A., Ono T., Hayashi M. (2017). Osteocyte regulation of orthodontic force-mediated tooth movement via RANKL expression. Sci Rep.

[24] Ohori F., Kitaura H., Marahleh A. (2019). Effect of TNF-α-induced sclerostin on osteocytes during orthodontic tooth movement. J Immunol Res.

[25] Ogawa S., Kitaura H., Kishikawa A. (2019). TNF-α is responsible for the contribution of stromal cells to osteoclast and odontoclast formation during orthodontic tooth movement. PloS One.

[26] Qi J., Kitaura H., Shen W.R. (2019). Establishment of an orthodontic retention mouse model and the effect of anti-c-Fms antibody on orthodontic relapse. PloS One.

[27] Noguchi T., Kitaura H., Ogawa S. (2020). TNF-α stimulates the expression of RANK during orthodontic tooth movement. Arch Oral Biol.

[28] Kitaura H., Sands M.S., Aya K. (2004). Marrow stromal cells and osteoclast precursors differentially contribute to TNF-alpha-induced osteoclastogenesis in vivo. J Immunol.

[29] Kitaura H., Zhou P., Kim H.J. (2005). M-CSF mediates TNF-induced inflammatory osteolysis. J Clin Invest.

[30] Metzger C.E., Narayanan A., Zawieja D.C., Bloomfield S.A. (2017). Inflammatory bowel disease in a rodent model alters osteocyte protein levels controlling bone turnover. J Bone Miner Res.

[31] Kim J.H., Lee D.E., Cha J.H., Bak E.J., Yoo Y.J. (2014). Receptor activator of nuclear factor-κB ligand and sclerostin expression in osteocytes of alveolar bone in rats with ligature-induced periodontitis. J Periodontol.

[32] Lowney J.J., Norton L.A., Shafer D.M., Rossomando E.F. (1995). Orthodontic forces increase tumor necrosis factor alpha in the human gingival sulcus. Am J Orthod Dentofacial Orthop.

[33] Jayaprakash P.K., Basavanna J.M., Grewal H. (2019). Elevated levels of Interleukin (IL)-1β, IL-6, tumor necrosis factor-α, epidermal growth factor, and β2-microglobulin levels in gingival crevicular fluid during human Orthodontic tooth movement (OTM). J Fam Med Prim Care.

